# Increased Levels of Sphingosylphosphorylcholine (SPC) in Plasma of Metabolic Syndrome Patients

**DOI:** 10.1371/journal.pone.0140683

**Published:** 2015-10-14

**Authors:** Nahed El-Najjar, Evelyn Orsó, Stefan Wallner, Gerhard Liebisch, Gerd Schmitz

**Affiliations:** Institute of Clinical Chemistry and Laboratory Medicine, University Hospital Regensburg, Regensburg, Germany; Medical University of South Carolina, UNITED STATES

## Abstract

Recent developments in lipid mass spectrometry enable extensive lipid class and species analysis in metabolic disorders such as diabesity and metabolic syndrome. The minor plasma lipid class sphingosylphosphorylcholine (SPC) was identified as a ligand for lipid sensitive G-protein coupled receptors playing a key role in cell growth, differentiation, motility, calcium signaling, tissue remodeling, vascular diseases and cancer. However, information about its role in diabesity patients is sparse. In this study, we analyzed plasma lipid species in patients at risk for diabesity and the metabolic syndrome and compared them with healthy controls. Our data show that SPC is significantly increased in plasma samples from metabolic syndrome patients but not in plasma from patients at risk for diabesity. Detailed SPC species analysis showed that the observed increase is due to a significant increase in all detected SPC subspecies. Moreover, a strong positive correlation is observed between total SPC and individual SPC species with both body mass index and the acute phase low grade inflammation marker soluble CD163 (sCD163). Collectively, our study provides new information on SPC plasma levels in metabolic syndrome and suggests new avenues for investigation.

## Introduction

Metabolic syndrome (MetS) is characterized by a combination of different metabolic abnormalities including abdominal obesity, dyslipidemia, increased fasting glucose, and hypertension [1]. These metabolic disorders collectively lead to more complex diseases including type-2 diabetes (T2D) and cardiovascular diseases (CVD) [1]. Several lines of evidence support the role of lipids in metabolic health. The advancement of plasma lipidomic analysis allowed the identification of numerous lipid classes and species as surrogate markers for age-related diseases, including hypertension, T2D, and CVD [1–3]. Previous studies have reported the association of several lipid molecules such as cholesteryl esters (CE 18:2, CE 16:0), the phosphatidylethanolamine glycerophospholipid species (PE 36:2), and the diacylglycerol species (DG 36:2, DG 34:0) with MetS risk factors [1,4,5]. Other plasma lipids such as phosphatidylinositol (PI), phosphatidylglycerol (PG), and PE were found to be positively associated with prediabetes and T2D [6]. Furthermore, plasma ceramides (Cer), the most extensively studied sphingolipids [7,8], have been shown to be positively correlated with both T2D and prediabetes [6]. Moreover, a positive association between Cer species (Cer 16:0, Cer 24:1) and cardiovascular mortality has been established [9]. So far with the help of electrospray ionization tandem mass spectrometry (ESI-MS/MS) the involvement of minor sphingolipids such as sphingosylphosphorylcholine (SPC) species has been unraveled. SPC is a naturally occurring plasma lipid with a concentration estimated at around 50±15nM [10,11]. The source of plasma SPC is unclear but it can be released from activated platelets [12]. As SPC is also an important constituent of lipoproteins in plasma [11]; thus, it is not surprising that it can affect both cardiac and blood vessel function. SPC acts as a ligand for G-protein coupled receptors namely pertussis toxin sensitive receptors which may involve G_αi_, and G protein-associated Edg receptors of sphingosine-1-phosphate (S1P) (Edg-1/S1P1, Edg-5/S1P2, Edg-3/S1P3) as well as lysphosphatidic acid (LPA) receptors (Edg receptors (Edg-2/LPA1, Edg-4/LPA2, Edg-7/LPA3) [13,14]. Consequently, SPC, by acting as ligand for the aforementioned receptors as well as to other not-yet identified receptors, regulates several cellular processes including cell growth, differentiation, motility, apoptosis, and calcium levels [15,16]. SPC has been reported to induce lung tissue remodeling [17] and cell death in non-small lung cancer cells [18]. It also plays a key role in the migration/invasion of pancreatic cancer cells [19] and promotes vascular dysfunction [20]. However, *in vivo* evidences of the molecular mechanisms of SPC-induced vascular dysfunction are limited. SPC may contribute to the pathogenesis of vascular diseases not only through the modulation of the vascular tone but also by inducing the transformation of the proliferation and migration potential of vascular smooth muscle cells [14], as well as by acting as a pro-inflammatory mediator [14]. Despite the lack of studies showing a significant increase in SPC plasma levels in diseases, a significant elevation in its levels was found in dried blood spots from Niemann-Pick B (NPB) patients and in plasma of Niemann-Pick C (NPC) patients [21]; it was proposed thereafter as a potential biomarker for Niemann-Pick diseases [22].

The alterations of plasma lipid levels especially through lipid hydrolysis that generates minor lipid mediators render them attractive as clinical tools for risk assessment and monitoring of disease progression. Since SPC exerts its effect through binding to G-protein coupled receptors, already identified on the plasma membrane of many cell types [23,24], an elevation in its plasma levels may lead to the deterioration of cell function and dysregulation of vascular remodeling. Nevertheless, the relation of lipids with obesity and low levels of inflammation, as hallmarks of MetS [25] is well acknowledged. Besides C-reactive protein, the concentration of the low-grade inflammation marker sCD163 is increased in obese and T2D patients and also predicts increased risk of T2D [25,26]. sCD163 is also found, independently of conventional risk factors, as a significant predictor of coronary atherosclerosis [27]. Therefore, the aim of this study was 1) to analyze the changes in plasma lipid profile of patients at risk for diabesity and with a full blown MetS with specific emphasis on SPC during the evolution of these pathological states, and 2) to correlate plasma lipid levels with body mass index and the low-grade inflammation marker sCD163.

## Subjects and Methods

### Study population

The study was approved (Proposal 08/119) by the Ethics Committee of the Faculty of Medicine of the University Hospital of Regensburg. Prior to participation written informed consent was obtained from all participants. Recruited age matched male subjects (n = 64) were divided into healthy controls (n = 12), patients at risk for diabesity (n = 19), and MetS patients (n = 33) depending on the WHO criteria [28]. MetS patients had at least three out of the five WHO criteria: central obesity (BMI>30kg/(m)2), elevated triglycerides (TG) (150 mg/dl), increased fasting plasma glucose (100 mg/dl), along with a decrease in HDL levels (40mg/dl). Risk patients were those who had less than three criteria and healthy controls did not have any criteria. Characteristics of the subjects are presented in **Table 1**.

**Table 1:** Characteristics of the study groups. As expected, BMI, plasma glucose, triglycerides, HDL-cholesterol, VLDL-cholesterol and sCD163 are significantly different between healthy controls and MetS patients.

Male Subjects	Control (n = 12)	Risk (n = 19)	MetS (n = 33)	Ctr vs. Risk	Ctr vs. MetS	Risk vs. MetS
Characteristic	Median ± SE	Median ± SE	Median ± SE	*P* value	*P* value	*P* value
Age (years)	47.4 ± 2.3	48.5 ± 2.3	54.9 ± 1.8	0.98	0.051	0.09
BMI (Kg/(m)2)	23.6 ± 0.7	26.3 ± 0.8*	33.2 ± 1.2*** ≠≠≠	0.042	<0.0001	<0.0001
Glucose (mg/dl)	90.9 ± 2.4	99.9 ± 2.3*	119.6 ± 9.9*	0.033	0.025	0.173
Triglycerides (mg/dl)	84.6 ± 8.9	118.6 ±10.8	194.5 ± 14.5*** ≠≠≠	0.062	<0.0001	<0.0001
Total Cholesterol (mg/dl)	161.9 ± 4.5	165.4 ± 2.3	162.0 ± 2.1	0.865	1.000	0.616
LDL Cholesterol (mg/dl)	123.9 ±11.5	129.1 ± 8.1	116.0 ± 6.2	0.976	0.905	0.498
HDL Cholesterol (mg/dl)	64.4 ± 4.5	56.0 ± 3.9	42.0 ± 2.2** ≠	0.412	0.001	0.011
VLDL Cholesterol (mg/dl)	19.5 ± 3.2	21.9 ± 5.2	38.1 ± 2.7*** ≠	0.972	<0.0001	0.029
sCD163 (μg/ml)	1.4 ± 0.2	1.6 ± 0.2	2.2 ± 0.2*	0.647	0.005	0.071
Adiponectin (μg/ml)	9.1 ± 1.1	8.4 ± 1.3	7.6 ± 0.6	0.972	0.561	0.911

BMI: Body Mass Index, LDL: low density lipoprotein, HDL: high density lipoprotein, VLDL: very low density lipoprotein.

*vs. control

* *p<*0.05

** *p<*0.001

*** *p<*0.0001

≠ vs. risk: ≠ *p<*0.05

≠ ≠ ≠ *p<*0.0001.

### Clinical chemistry analysis

A standard clinical chemistry analyzer (ADVIA-1800, Siemens) was used to determine the levels of cholesterol, triglycerides, HDL-cholesterol, LDL-cholesterol, and VLDL-cholesterol.

### Lipid analysis

Extraction of lipids was performed in the presence of non-naturally occurring lipid species as internal standards following the protocol described by Bligh and Dyer [29]. Plasma lipid species determination was completed using direct flow injection ESI-MS/MS in positive ion mode as described previously [30,31]. A precursor ion of *m/z* 184 was used for phosphatidylcholine (PC), lysophosphatidylcholine (LPC), and sphingomyelin (SM) [30,32]. A fragment ion of *m/z* 264 was used to analyze Cer while a fragment ion of *m/z* 369 was used for the analysis of free cholesterol (FC) and CE after selective derivatization of FC [33,31]. PE and PI were analysed following neutral loss fragment of 141 and 277 Da, respectively [34,35]. The analysis of PE-based plasmalogens was done as described by Zemski-Berry [36]. On the other hand, SPC and sphingosine-1-P (S1P) analysis was done using our previously established liquid chromatography-tandem mass spectrometry (LC-MS/MS) protocol [11,37]. Self programmed Excel Macros were used for data analysis of all lipids [30,38]. Lipid species were annotated according to the LipidomicNet proposal for shorthand notation of lipid structures derived from mass spectrometry [39]. Glycerophospholipid species annotation was based on the assumption of even-numbered carbon chains only. Sphingomyelin species were assigned based on the assumption of a sphingoid base with 2 hydroxyl groups.

### Statistical analysis

Results are expressed as means ± standard errors (SE). Statistical analysis was performed using IBM SPSS 20 Software Package. Comparisons between the different groups were evaluated using ANOVA followed by Dunnett *test*. Linear relationships were studied using *Pearson’s* correlation coefficient. The level of significance was set at 0.05.

## Results

### Plasma lipid profile in patients at risk for diabesity and MetS

**Fig 1:**
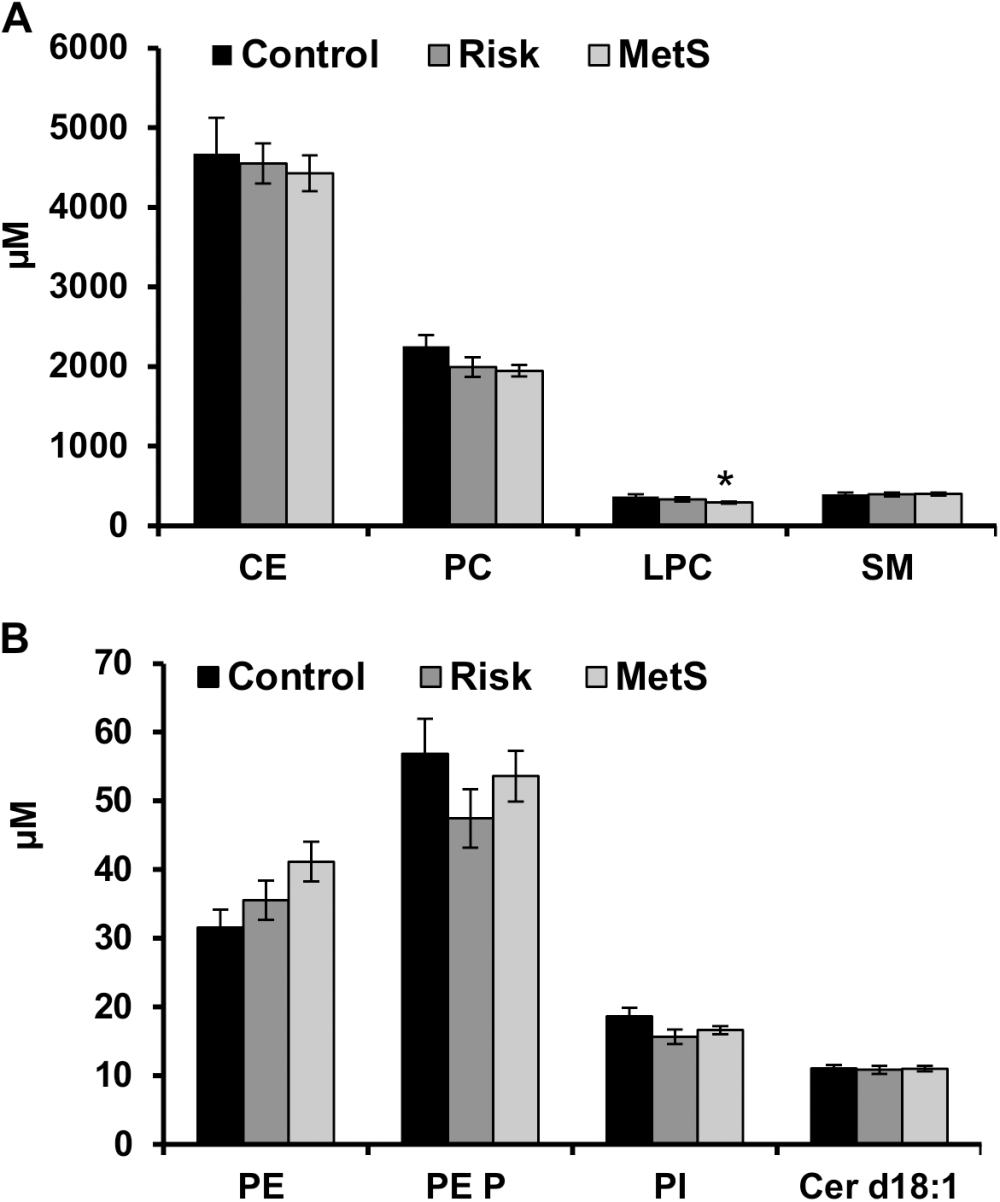
Plasma levels of CE, PC, LPC, SM (A), and PE, PE P, PI, and Cer (B) from controls and patient groups. From all the major lipid classes analyzed only changes in LPC levels reached significance in MetS patients ((n = 12 (Control), n = 19 (Risk), and n = 33 (MetS)) (A). Lipid species were analyzed either by LC-MS/MS or ESI-MS/MS, as described in Methods, and are expressed in μmol/l of plasma. Data presented as mean ± SEM. * *p*<0.05, statistically different with respect to control.

**Fig 2:**
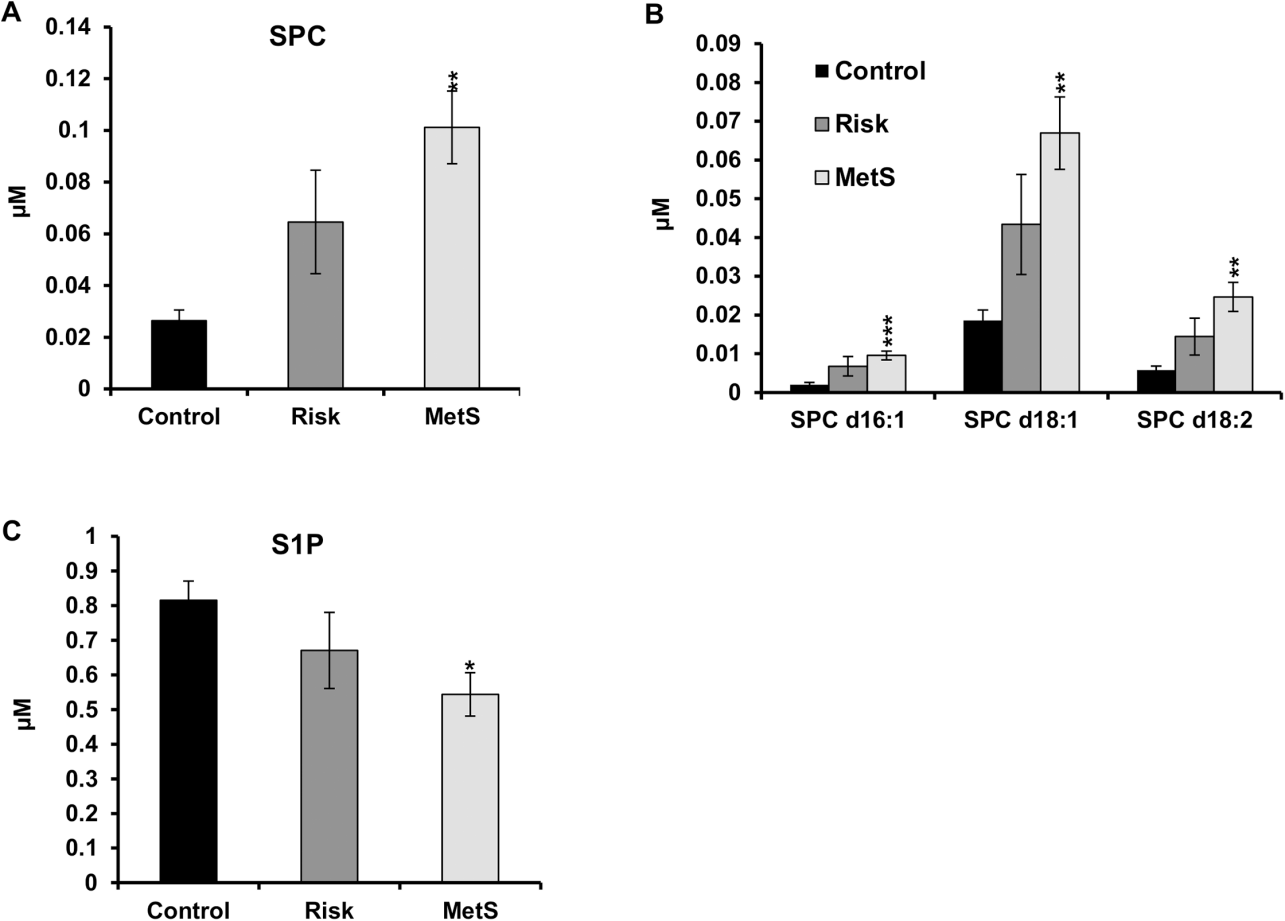
Plasma levels of SPC and S1P from controls and patient groups. (A): Total SPC; (B): SPC-species; (C): S1P. SPC levels were significantly increased in MS patients but not in those at risk for diabesity (A). SPC increase was due to an increase in all three main subspecies (B). MetS patients have significant lower levels of S1P (C). S1P and SPC-species (n = 8 (Control), n = 8 (Risk), and n = 9 (MetS) were analyzed by LC-MS/MS and are expressed in μmol/l of plasma. Data presented as mean ± SEM. * *p*<0.05, **p<0.005, ***p<0.0005 statistically different with respect to control.

Plasma lipid species levels from all groups (control, risk, and MetS) were analyzed using previously published ESI-MS/MS or LC-MS/MS procedures as described in the methods section. Figs 1 and 2 depict the changes observed in major lipid classes (CE, PC, SM, and LPC), and other less abundant lipid classes detected at levels below 50μM (Cer, PI, PE, PE P, and SPC). Interestingly, while the alterations observed in total levels of the majority of lipid classes did not reach significance, total levels of SPC and LPC were significantly different between the different groups (Figs 1A and 2A). In order to decipher the specific species responsible for the observed alterations, plasma from the studied patients was further analyzed and the levels of SPC- and LPC-species were determined.

The most significant difference was seen in the levels of SPC species. As compared to controls the total SPC levels in the plasma of MetS patients were significantly elevated by 3.8-fold (*p*<0.001) (Fig 2A) and associated with a similar increase in all detected SPC species i.e. SPC d16:1 (4.7-fold, *p*<0.0001), SPC d18:1 (3.6-fold, *p*<0.001), and SPC d18:2 (4.3-fold, *p*<0.001) (Fig 2B). However, the increase of total and individual SPC species in the plasma of patients at risk for diabesity did not reach significance (Fig 2A and 2B).

The level of S1P, which either originates from ceramide under conditions of increased sphingosine levels or from SPC under the action of autotaxin or other ecto-lysosphingomyelinase(s), was also measured in the plasma of all groups. Even though the plasma level of S1P is decreased in patients at risk for diabesity as compared to controls, it only reached significance (1.5-fold, *p*<0.05) in MetS patients (Fig 2C).

**Fig 3:**
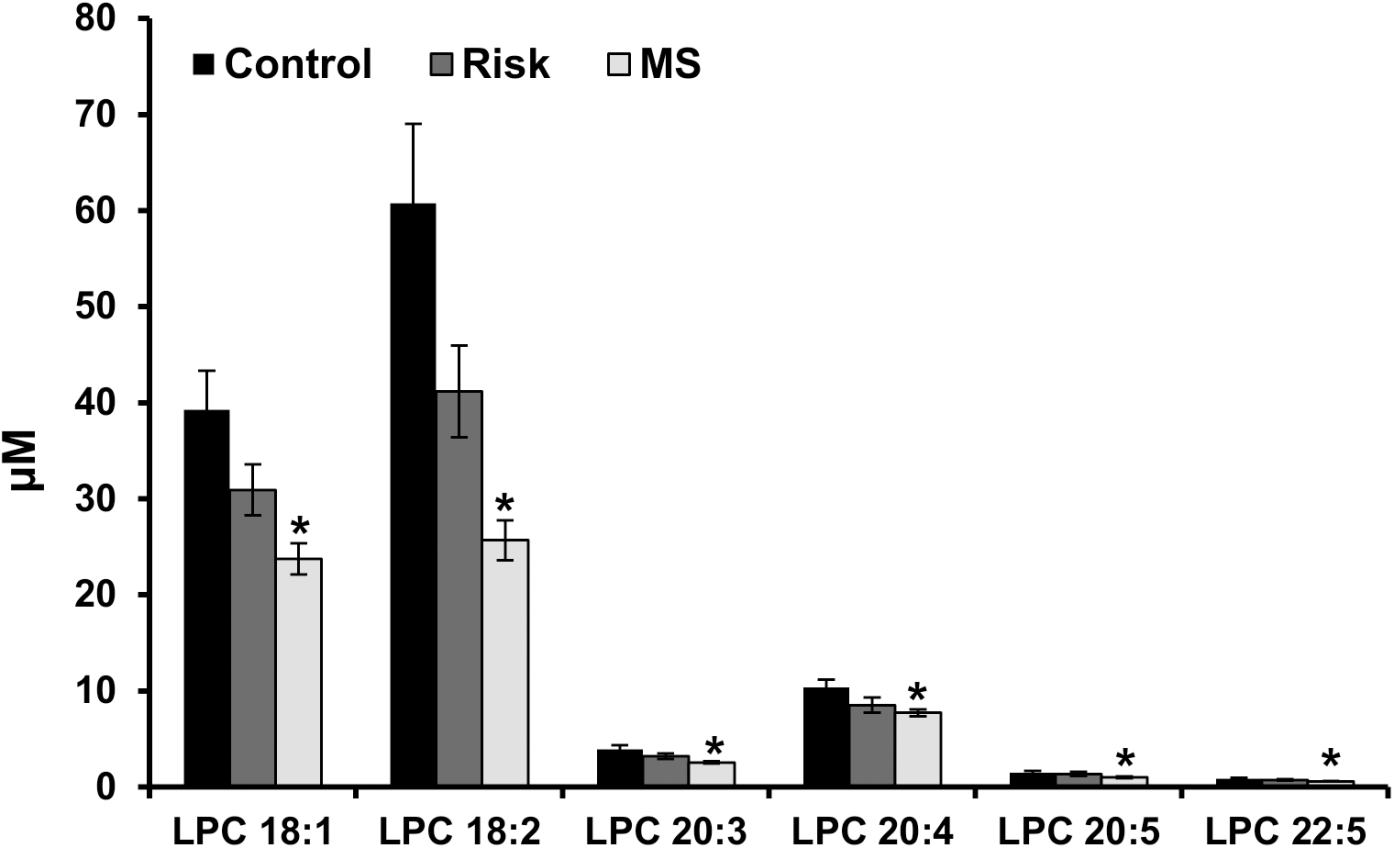
Plasma LPC species levels in controls and patient groups. A significant decrease in specific LPC species plasma levels was observed in MetS patients but not in patients at risk for diabesity ((n = 12 (Control), n = 19 (Risk), and n = 33 (MetS)). LPC-species were analyzed by ESI-MS/MS and are expressed in μmol/l of plasma. Data presented as mean ± SEM. * *p*<0.05, statistically different with respect to control.

Albeit the decrease in total plasma levels of phosphatidylcholine (PC) did not reach significance between the different groups, significant alterations in LPC levels were observed. When compared to healthy subjects the plasma levels of LPC were significantly decreased in MetS patients (*p*<0.05) (Fig 1A). The observed decrease was due to a pronounced decrease in individual LPC species i.e. LPC 18:1; LPC 18:2; LPC 20:3; LPC 20:4; LPC 20:5; LPC 22:5 (*p*<0.05) (Fig 3) while changes in the levels of the other detected species were not significant (Data not shown). On the other hand, neither total nor individual LPC species were affected in patients at risk for diabesity.

### Correlation of SPC, S1P and LPC with obesity and inflammation

The relation between lipids, obesity, low levels of inflammation and MetS is established [25]. MetS is strongly associated with increased risk of T2D and CVD. Therefore, we tried to elucidate in our patient groups the relation of SPC, S1P and LPC with both BMI and the low grade inflammation marker sCD163. Table 2 shows a strong significant positive correlation of BMI with total SPC (*r* = 0.694, *p*<0.0001) and individual species SPC d16:1 (*r* = 0.573, *p* = 0.004), SPC d18:1 (*r* = 0.693, *p*<0.0001), and SPC d18:2 (*r* = 0.724, *p*<0.0001). Also total SPC (*r* = 0.422, *p* = 0.045), SPC d18:1 (*r* = 0.429, *p* = 0.041), and SPC d18:2 (*r* = 0.439, *p* = 0.036) species correlated significantly with sCD163 while no correlation existed with SPC d16:1 (Table 2). In contrast to SPC, S1P showed a significant negative correlation with BMI (*r* = -0.422, *p* = 0.045) while the correlation with sCD163 did not reach significance (Table 3). A significant negative correlation was also observed between several LPC species and both BMI and sCD163 (Table 4). Whereas total LPC did not significantly correlate with BMI, a significant negative correlation between 6 LPC species and BMI was observed for LPC 18:1 (*r* = -0.334, *p* = 0.009), LPC 18:2 (*r* = -0.420, *p* = 0.001), LPC 18:3 (*r* = -0.326, *p* = 0.011), LPC 20:3 (*r* = -0.266, *p* = 0.040), LPC 20:5 (*r* = -0.279, *p* = 0.031), and LPC 22:5 (*r* = -0.292, *p* = 0.024). Interestingly, a positive correlation existed between BMI and LPC 22:4 (*r* = 0.283, *p* = 0.029). On the other hand, total LPC and 13 out of 15 LPC species negatively correlated with sCD163: LPC Total (*r* = -0.385, *p* = 0.002), LPC 15:0 (*r* = -0.334, *p* = 0.009), LPC 18:0 (*r* = -0.303, *p* = 0.019), LPC 18:1 (*r* = -0.307, *p* = 0.017), LPC 18:2 (*r* = -0.354, *p* = 0.006), LPC 18:3 (*r* = -0.274, *p* = 0.034), LPC 20:0 (*r* = -0.258, *p* = 0.046), LPC 20:3 (*r* = -0.421, *p* = 0.001), LPC 20:4 (*r* = -0.525, *p*<0001), LPC 20:5 (*r* = -0.313, *p* = 0.015), LPC 22:4 (*r* = -0.445, *p*<0001), LPC 22:5 (*r* = -0.580, *p*<0001), and LPC 22:6 (*r* = -0.473, *p*<0001). Comparison of the different species showed that unsaturated LPC species including mono- and polyunsaturated species but not saturated LPC species negatively correlated with BMI and sCD163 (Table 4).

**Table 2:** Correlation between BMI, sCD163 and triglycerides with total and individual SPC species.

Correlations	BMI	BMI	sCD163	sCD163	Triglyceride	Triglyceride	HDL	HDL
	R value	P value	R Value	P Value	R value	P value	R value	P value
SPC Total	.694**	<0001	.422*	.045	.480*	.020	-.587**	.004
SPC d16:1	.573**	.004	.305	.158	.529**	.009	-.576**	.005
SPC d18:1	.693**	<0001	.429*	.041	.465*	.025	-.580**	.005
SPC d18:2	.724**	<0001	.439*	.036	.481*	.020	-.589**	.004

*. Correlation is significant at the 0.05 level (2-tailed), and

**. Correlation is significant at the 0.01 level (2-tailed).

**Table 3:** Correlation between BMI and sCD163 with S1P.

S1P		
	R Value	P Value
BMI	-.422*	.045
sCD163	-.412	.051

*. Correlation is significant at the 0.05 level (2-tailed).

**Table 4:** Correlation between BMI and sCD163 total and individual LPC species.

LPC Species	BMI	BMI	sCD163	sCD163
	R Value	P value	R Value	P Value
LPC 15:0	-.128	.331	-.334**	.009
LPC 16:0	-.019	.887	-.205	.116
LPC 16:1	-.177	.176	-.145	.268
LPC 18:0	.039	.769	-.303*	.019
LPC 18:1	-.334**	.009	-.307*	.017
LPC 18:2	-.420**	.001	-.354**	.006
LPC 18:3	-.326*	.011	-.274*	.034
LPC 20:0	.013	.920	-.258*	.046
LPC 20:3	-.266*	.040	-.421**	.001
LPC 20:4	-.160	.221	-.525**	<0001
LPC 20:5	-.279*	.031	-.313*	.015
LPC 22:4	.283*	.029	-.445**	<0001
LPC 22:5	-.292*	.024	-.580**	<0001
LPC 22:6	-.224	.086	-.473**	<0001
LPC Total	-.165	.207	-.385**	.002
LPC Saturated	-.002	.987	-.237	.068
LPC Unsaturated	-.384**	.002	-.378**	.003
LPC Monounsaturated	-.326*	.011	-.298*	.021
LPC Polyunsaturated	-.398**	.002	-.403**	.001

*. Correlation is significant at the 0.05 level (2-tailed), and

**. Correlation is significant at the 0.01 level (2-tailed).

## Discussion

High levels of toxic lipid intermediates play a major role in the progression of many diseases including obesity, type-2 diabetes (T2D), and cardiovascular diseases (CVD) [40]. The aim of this study was to monitor changes in the plasma lipidome of patients at risk for diabesity and MetS patients and to evaluate the contribution of these changes to the evolution of disease pathology. The classification of the subjects used in this study was based on the WHO guidelines and is summarized in Table 1.

In accordance to our previously published data [41], this study shows that vs. controls the alterations of PC, SM, PE, PE P, and Cer levels did not reach significance in the plasma of MetS patients. Our data related to Cer levels are in contrast to the data presented by Meikle *et al*., [6], showing a significant increase in the levels of Cer in prediabetes and T2D. However, the average age of prediabetes patients used in the former study was 69 (58–74) years which differed from the average age of the patients at risk for diabesity and MetS in our study which was 48.5 ± 2.3 and 54.9 ± 1.8 years, respectively. Furthermore, the size of our study groups is significantly smaller than that of Meikle *et al*., [6]. Consequently, the observed discrepancy may, therefore, be age and/or study size dependent.

Glycerophospholipids and their metabolites lysophospholipids (LPLs) are involved in signal transduction mechanisms regulating cell proliferation and apoptosis, as well as in disease development [42,43]. LPLs are generated by the enzymatic reaction of members of the phospholipases A2 family [44] and by the action of lecithin-cholesterol acyltransferase (LCAT) activity [45]. While the decrease in PC levels did not reach significance, a significant decrease of LPC plasma levels was observed in MetS patients. The widespread reduction in LPC species in the plasma of MetS individuals was not surprising as we [41] and others [46] have previously reported a similar trend. Similarly to our earlier findings [41], this study shows a significant negative correlation between individual LPC species and both BMI and the low grade inflammatory marker sCD163 (Table 4), further supporting a potential anti-inflammatory role of LPC in diabesity and MetS patients.

**Fig 4:**
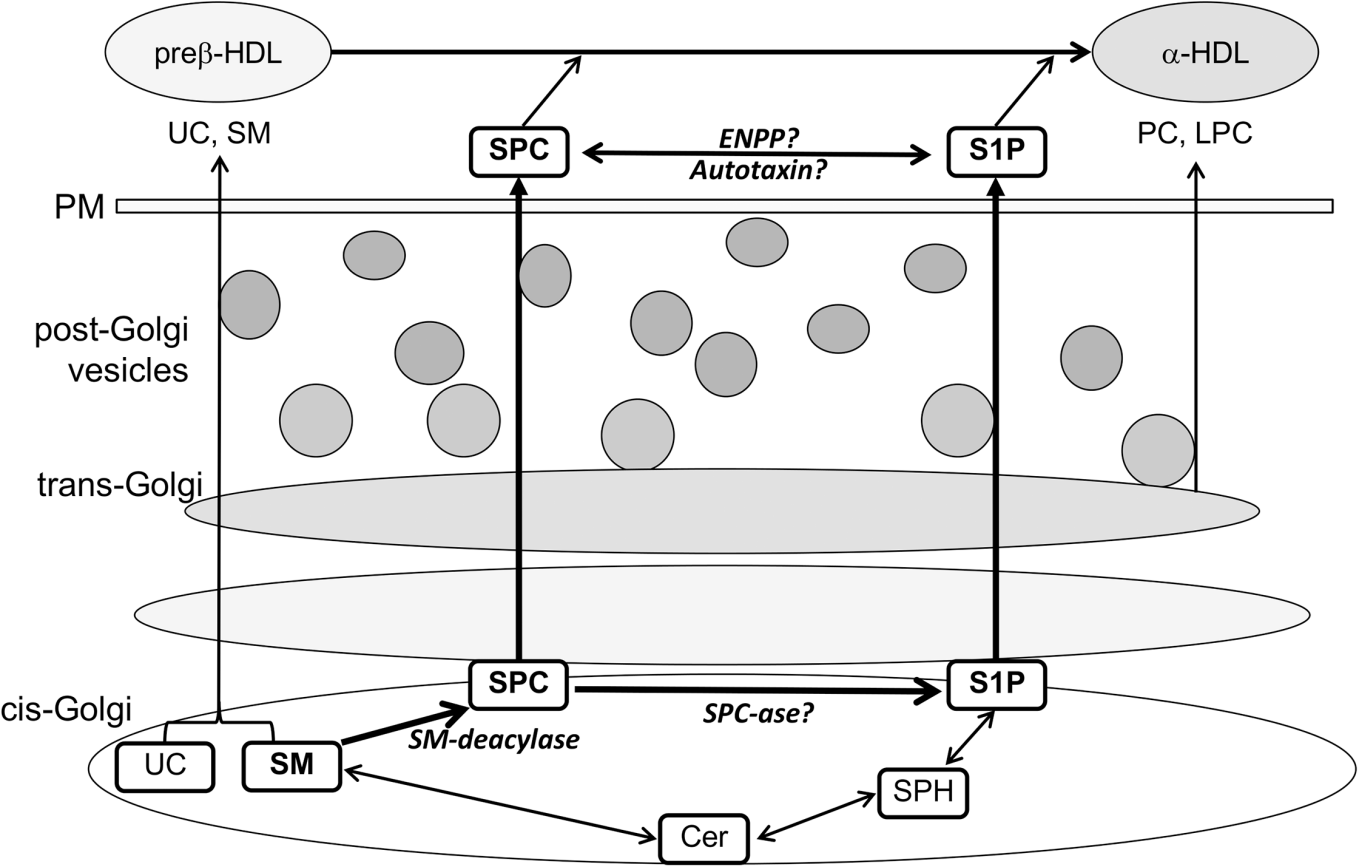
Hypothetical scheme depicting SPC and S1P synthesis. Ceramides (Cer) are generated from the sphingomyelinase pathway and other pathways such as the salvage pathway and the de novo synthesis pathway (not shown in this scheme). Sphingosine (SPH) which can be formed from the degradation and recycling of complex sphingolipids and glycosphingolipids in an acidic environment (salvage pathway) may also contribute to Cer metabolism. SPH is phosphorylated by SPH kinase to sphingosine-1-phosphate (S1P), a bioactive lipid intermediates with several effects. In addition to Cer, SPC is another biologically active lipid metabolite generated from sphingomyelin (SM) under the action of a SM-deacylase. Secreted SPC is likely a substrate for autotaxin (ATX), an exoenzyme with lysophospholipase D activity, which leads to S1P generation. Alternatively, S1P can be converted from SPC by ectonucleotide pyrophosphatase/phosphodiesterases (ENPPs). SPC and S1P can both induce their effects through binding to G-protein coupled receptors present on different cell types. It cannot be excluded that an intracellular SPC—> S1P conversion also occurs by a yet unidentified SPC-ase, and not SPC but rather S1P is secreted from cells predominantly. Extracellular S1P might be converted back to SPC through not yet identified enzyme(s). Substantial amounts of extracellular SPC and S1P are loaded to preβ-HDL particles and therefore they may contribute to the composition and/or maturation of α-HDL lipoproteins. UC: unesterified cholesterol, PC: phosphatidylcholine, LPC: lysophosphatidylcholine, PM: plasma membrane.

In the last 10 years more than 150 articles have been published on SPC but none reported its modulation in the plasma of MetS patients. Therefore, to our knowledge this is the first study demonstrating a significant increase in SPC levels in the plasma of MetS patients (3.8-fold vs. control, *p*<0.001). Detailed analysis showed that all detected species SPC d16:1, SPC d18:1 and SPC d18:2 were increased by more than 3.5-fold in the plasma of MetS patients with respect to their normal counterpart. SPC occurs naturally in plasma at a concentration of 50±15nM [10,11]. SPC is also generated intracellularly from SM under the action of SM deacylase (Fig 4). An increase in SPC levels could represent a spillover mechanism upon increased SM hydrolysis or from a decrease in SPC metabolism. SPC is metabolized to sphingosine-1-phosphate (S1P) by autotaxin (ATX), an exoenzyme with lysophospholipase D activity, or by other ectonucleotide pyrophosphatase/phosphodiesterases (ENPPs) [13,47,48]. The occurrence of ATX in the plasma suggests that SPC could be directly metabolized to S1P [48]. ATX is a multifunctional protein that has the ability to hydrolyze membrane or secreted glycerophospholipids to produce bioactive mediators such as LPA and S1P [13]. ATX is also established as an adipose derived secreted enzyme that controls adipose tissue expansion, brown adipose tissue function as well as energy expenditure [49]. A significant down-regulation in the expression of ATX was reported in the retina of diabetic rats and in human subcutaneous fat along with a significant reduction in its levels in the serum of obese patients [49]. Our study shows, a significant decrease in plasma S1P levels in MetS patients as compared to control subjects while no significant changes in the levels of plasma SM was observed. Therefore, an increase in SPC levels at least in part could be due to a decrease in its metabolism. The significantly lower levels of LPC species in MetS are likely the consequence of the lower levels of HDL-cholesterol in MetS and likewise to the decreased LCAT activity. The latter is a major determinant of plasma LPC levels. Interestingly, it is worth mentioning that although S1P and SPC are both constituents of HDL [11,14,50], the significant decrease in HDL levels observed in MetS patients (Table 1) inversely correlated with plasma SPC levels (Table 2) but showed no correlation with plasma S1P levels (*r* = 0.359, *p* = 0.101). As SPC might act through different types of receptors differentially distributed on many cell types [51], an increase in SPC plasma levels may lead to a defect in diverse cell functions in MetS patients. Since the studies on SPC plasma levels are scarce, a better understanding of the function of SPC might help to clarify the relevance of its elevation in the plasma of MetS patients. SPC is a pleiotropic lipid mediator involved in many physiological and pathological effects depending on the type of tissues and/or disease [14,52]. SPC plays a crucial role in calcium (Ca^2+^) regulation [15,16]. This function is of utmost importance for the function of the cardiovascular system. Consequently, any disturbance of this function may lead to disease development. SPC acts as an extracellular and cellular Ca^2+^ mediator [51]. This has been proven by the fact that extracellular application of SPC leads to a rapid increase in intracellular Ca^2+^ in different cell types [15,16]. The detrimental effects of a sustained increase in Ca^2+^ levels are well known. While a transient increase in cytosolic Ca^2+^ is necessary for cell function such as contraction in contractile cells per se a constitutive elevation of Ca^2+^ is pathogenic. For instance, a sustained increase in intracellular Ca^2+^ levels leads to the switching of vascular smooth muscle (VSMC) cells from contractile to synthetic phenotype which is a hallmark of atherosclerosis [53]. Interestingly, SPC induces the proliferation and migration of VSMC in the nM range, an important effect that leads to the formation of a neointima in blood vessels [14]. SPC also induces the contraction of VSMC [54] and plays a major role in the pathogenesis of abnormal contraction of cerebral arteries [20,55]. It has been recently reported that the extent of SPC induced VSMC contraction correlates with total plasma cholesterol [20]. In this study we did not see a correlation between SPC and cholesterol as the levels of this latter one were not significantly different between the different groups (Table 1), probably since some of the MetS patients are under lipid lowering therapy. On the other hand, total and individual SPC species strongly correlated with TG levels (Table 2). In addition to the stimulatory effects of SPC on proliferation, migration, and contraction of VSMC, SPC has also been found to act as a pro-inflammatory mediator in the vascular system. SPC increased the release of the inflammatory protein chemokine monocyte chemoattractant protein-1 (MCP-1), both in cultured VSMC and in cerebral arteries [12]. Addition of SPC to VSMC induced also the release of TNF-α, an inflammatory cytokine involved in cardiovascular disease [14]. This study shows a significant correlation between SPC and the acute phase response in low grade inflammation marker sCD163 (Table 2), a significant predictor of coronary atherosclerosis [27]. Nonetheless, the precise molecular mechanism of SPC in acute phase response is unclear and merits further investigation.

Finally, it is noteworthy to mention that changes observed with the lipid classes in the plasma of patients at risk for diabesity did not reach significance. Based on this finding one could speculate that a significant increase in TG and sCD163 and a decrease in HDL are essential to induce significant changes in plasma lipids profile. This is further supported by our findings showing that SPC correlates significantly with TG and sCD163, and inversely correlates with HDL (Table 2).

## Conclusion

Lipidomic analysis of major and minor lipid classes was performed by mass spectrometry in order to gain more insights into the changes affecting circulating lipids in the plasma of patients at risk for diabesity and MetS. This study confirms and extends previous findings on LPC levels in obese and MetS patients and shows, for the first time, a significant increase in SPC plasma levels in MetS patients. Our data show, collectively, that increased plasma SPC levels along with its strong correlation with BMI and sCD163 may be a reporter for the progression of the disease associated with inflammation and the risk of cardiovascular dysfunction. The fact that there were no significant changes in the plasma levels of SM and Cer, alterations already observed in T2D, underlines the significance of our findings on SPC as a potential early biomarker. Future studies in larger cohorts are needed to 1) better understand why SPC is increased in MetS patients, and 2) investigate its potential as a biomarker for MetS-associated cardiovascular dysfunction and T2D.

## Supporting Information

S1 File Plasma lipidomic analysis form controls and patients groups used in the study. (XLSX)
